# Conversion of organosolv pretreated hardwood biomass into 5-hydroxymethylfurfural (HMF) by combining enzymatic hydrolysis and isomerization with homogeneous catalysis

**DOI:** 10.1186/s13068-021-02022-9

**Published:** 2021-08-28

**Authors:** Grigorios Dedes, Anthi Karnaouri, Asimina A. Marianou, Konstantinos G. Kalogiannis, Chrysoula M. Michailof, Angelos A. Lappas, Evangelos Topakas

**Affiliations:** 1 Industrial Biotechnology & Biocatalysis Group, School of Chemical Engineering, National Technical University of Athens, Zografou Campus, 9 Iroon Polytechniou Str, 15780 Athens, Greece; 2 Biochemical Process Engineering, Chemical Engineering, Department of Civil, Environmental and Natural Resources Engineering, Luleå University of Technology, 97187 Luleå, Sweden; 3 Center for Research and Technology Hellas, Chemical Process and Energy Resources Institute, 57001 Thessaloniki, Greece

**Keywords:** 5-hydroxymethylfurfural, Lignocellulosic biomass, Isomerization, Homogeneous catalysis

## Abstract

**Background:**

Over the last few years, valorization of lignocellulosic biomass has been expanded beyond the production of second-generation biofuels to the synthesis of numerous platform chemicals to be used instead of their fossil-based counterparts. One such well-researched example is 5-hydroxymethylfurfural (HMF), which is preferably produced by the dehydration of fructose. Fructose is obtained by the isomerization of glucose, which in turn is derived by the hydrolysis of cellulose. However, to avoid harsh reaction conditions with high environmental impact, an isomerization step towards fructose is necessary, as fructose can be directly dehydrated to HMF under mild conditions. This work presents an optimized process to produce fructose from beechwood biomass hydrolysate and subsequently convert it to HMF by employing homogeneous catalysis.

**Results:**

The optimal saccharification conditions were identified at 10% wt. solids loading and 15 mg enzyme/g_solids_, as determined from preliminary trials on pure cellulose (Avicel® PH-101). Furthermore, since high rate glucose isomerization to fructose requires the addition of sodium tetraborate, the optimum borate to glucose molar ratio was determined to 0.28 and was used in all experiments. Among 20 beechwood solid pulps obtained from different organosolv pretreatment conditions tested, the highest fructose production was obtained with acetone (160 °C, 120 min), reaching 56.8 g/100 g pretreated biomass. A scale-up hydrolysis in high solids (25% wt.) was then conducted. The hydrolysate was subjected to isomerization eventually leading to a high-fructose solution (104.5 g/L). Dehydration of fructose to HMF was tested with 5 different catalysts (HCl, H_3_PO_4_, formic acid, maleic acid and H-mordenite). Formic acid was found to be the best one displaying 79.9% sugars conversion with an HMF yield and selectivity of 44.6% and 55.8%, respectively.

**Conclusions:**

Overall, this work shows the feasibility of coupling bio- and chemo-catalytic processes to produce HMF from lignocellulose in an environmentally friendly manner. Further work for the deployment of biocatalysts for the oxidation of HMF to its derivatives could pave the way for the emergence of an integrated process to effectively produce biobased monomers from lignocellulose.

**Supplementary Information:**

The online version contains supplementary material available at 10.1186/s13068-021-02022-9.

## Introduction

Today, the continually increasing worldwide demand for energy and chemicals necessitate the emergence of utilizing sustainable carbon sources able to support the production of numerous value-added compounds that can be employed as starting materials, precursors or building blocks for, among others, biofuels, polymers and pharmaceuticals. In this regard, lignocellulosic biomass is a potential candidate due to the fact that it is the most abundant renewable carbon source on the planet. In the past, lignocellulosic biomass has been reported as a promising feedstock for the production of biofuels, such as bioethanol and biodiesel [1–3]. However, since the biomass constituents can lead to the production of many other value-added products, along with the fact that large-scale bioethanol production faces some limitations, lignocellulose has started to expand as a raw material within the frame of the biorefinery concept [4]. One of these directions leads to the production of furans, mainly 5-hydroxymethylfurfural (HMF) and furfural (FA), which can be subsequently used as precursors for 2,5-furandicarboxylic acid (FDCA) and 2-furancarboxylic acid (FCA), thus driving the synthesis of biobased polymers [5]. FDCA is a biomass derived monomer that can function as a renewable building block for the production of polyethylene furanoate (PEF), a green alternative to its petrochemically produced counterpart polymer polyethylene terephthalate (PET). This process initially follows the typical lignocellulose biorefinery routes, namely, pretreatment and enzymatic saccharification, for the fractionation of biomass components and the glucose/xylose production, respectively. The monomeric sugars can be subsequently employed for the production of furans via chemocatalytic processes [6] or by employing the use of both enzymes and chemicals for the production of the intermediates and the final product [5, 7–9]. The introduction of enzymes in addition to chemicals in the process is a more attractive scenario, as it offers great regioselectivity and it is performed in milder conditions; therefore, it is considered environmentally friendly.

It is known that aldoses, such as glucose, can be effectively transformed into HMF, nevertheless, with the use of extreme conditions (i.e. high pressure, high temperature). On the contrary, using ketoses, such as fructose as the substrate, the HMF transformation can be achieved via a single chemical dehydration, rendering the process much milder [10]. For that reason, the efficient green valorization of lignocellulosic biomass for the production of monomers requires enzymatic saccharification to be followed by an isomerization step that can be performed by a glucose isomerase [11]. However, this isomerization reaction is halted by a thermodynamic equilibrium preventing the fructose formation in high yields. Increasing the yield of the process requires the addition of sodium tetraborate, which binds to fructose, therefore, effectively removing it from the reaction and shifting the equilibrium towards it. Hence, while the typical equilibrium stands at around 50%, with the addition of sodium tetraborate the fructose yield can increase at even 80% [12].

After isomerization of glucose to fructose, the latter can be used for the production of HMF via dehydration. Inorganic acids, such as HCl and H_2_SO_4_ as homogeneous catalysts, have demonstrated significant activity towards HMF production from sugars. In particular, using Ionic Liquids (IL), organic solvents or biphasic systems as the reaction medium and HCl or H_2_SO_4_ as catalyst, fructose converts efficiently to HMF reaching high yields ranging from 78 to 97% [13–16]. Nonetheless, these systems have drawbacks related to products challenging recovery and solvent recycling. On the other hand, water is an economical and environmentally friendly solvent, the use of which can overcome the above-mentioned obstacles. Therefore, acid-catalyzed conversion of fructose in aqueous medium has gained increased attention, with 65% being one of the higher HMF yields achieved so far, using H_3_PO_4_ as catalyst (pH 2) [17]. However, very few studies are focusing on a cascade process, combining enzymatic and chemocatalytic processes, for the synthesis of HMF from actual sugar-rich hydrolysates obtained from saccharification of lignocellulosic biomass [18].

In our previous work, we have developed OxiOrganosolv, an efficient pretreatment method to delignify the beechwood biomass [19], leaving behind a sugar-rich solid fraction that can be used for the production of furan derivatives. In the present study, an attempt to employ biocatalytic routes for the conversion of these organosolv pretreated samples to fructose is presented, by examining the effect of different pretreatment parameters on the final fructose yield. By integrating an acid-free organosolv biomass fractionation process together with high-gravity enzymatic hydrolysis for the saccharification of sugar streams and efficient enzymatic isomerization, a concentrated fructose syrup is obtained. Moreover, different homogeneous catalysts are evaluated as potential candidates for the efficient dehydration of sugars to furans. Through the use of numerous substrates and chemical catalysts, the results of this work prove that is possible to establish a process that ends in HMF production from real biomass samples.

## Results and discussion

### Screening of different biomass pretreatment processes for the production of fructose

Prior to hydrolysis of pretreated lignocellulosic biomass samples, preliminary screening of different initial solids concentration and enzyme loading was carried out to identify the optimal hydrolysis conditions that could maximize the cellulose conversion to glucose. Avicel® PH-101 (Sigma-Aldrich, U.S.) was used as a model substrate. The results, depicted at Additional file 1: Figure S1, indicate that % cellulose conversion yield remained high (72.9%) up to 10% wt. solids loading, while for higher concentration, it was decreasing, eventually reaching 49.8% for 16% wt. % solids. As far as the enzyme loading is concerned, it is evident that the increase in cellulose conversion between the 10 mg enzyme/g_solids_ and the 15 mg enzyme/g_solids_ is much greater than the respective one between 15 and 20 mg enzyme/g_solids_, with the respective increase being 15.4% and 8.7%. Furthermore, for enzyme loading higher than 20 mg/g_solids_, the increase in cellulose conversion is not as significant. Considering that the enzyme cost comprises a significant bottleneck in the overall economic integration of the process, the optimum enzyme concentration was set at 15 mg enzyme g_solids_ in all biomass experiments.

**Fig. 1:**
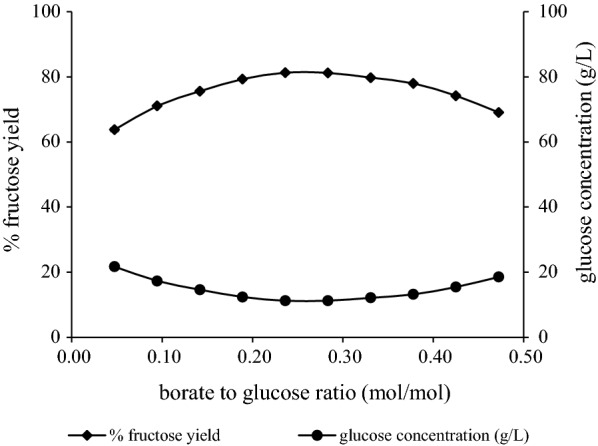
Study of the optimum borate loading for the isomerization reactions. Effect of different molar ratios of borate to glucose on the reduction of glucose concentration and the yield of the isomerization reaction

Similarly, the optimum ratio of borate to glucose leading to the highest yield during isomerization reaction was identified with pure glucose as substrate. As presented in Fig. 1 the maximum yield was observed for a molar ratio sodium tetraborate to glucose equal to 0.28, so this condition was used in all isomerization reactions with biomass hydrolysates. Other studies report that the borate to sugar molar ratio that maximizes the conversion yields lies within the range of 0.33–0.5 [18], or it is 0.5 [11]. In both cases, the results of our work indicate a lower demand in sodium tetraborate for effective isomerization, which is a significant factor to take into consideration as far as the economic integration of the process is concerned.

After the optimal hydrolysis conditions were defined, a number of different pretreated biomass samples were tested regarding both their susceptibility towards hydrolysis and the production of fructose. The different pretreatment conditions concerning the organic solvent employed (acetone—ACO, ethanol—EtOH, and tetrahydrofuran—THF), the residence time and temperature, as well as the oxygen pressure introduced are presented in Table 1, while the compositional analysis of the solid pulps obtained after pretreatment are described in Additional file 1: Table S1. The as such pretreated samples were subjected to enzymatic hydrolysis and isomerization and the respective yields of fructose are described in Table 2. It is evident that the cellulose-rich pulps obtained after organosolv fractionation were amenable to enzymatic saccharification, thus reaching cellulose to glucose conversion yields higher than 70% in most cases, compared to 25.9% conversion of the untreated feedstock. These results underline the efficiency of the OxiOrganosolv pretreatment process and verify the study previously reported by our group [19]. By employing organic solvents and mild oxidative conditions, OxiOrganosolv fractionation achieves efficient delignification and leads to solid pulps with high cellulose (even higher than 85 wt. %) and low lignin content (even lower than 2 wt. %), which can serve as starting materials for the production of value-added products through biocatalysis, fermentation [20, 21] and chemocatalytic processes. Removal of lignin is of pivotal importance in the production of furans from biomass, since non-specific adsorption of enzymes onto the substrate during saccharification is eliminated. In addition, the presence of phenolic compounds might interfere with the subsequent sugar dehydration step towards the production of HMF, thus causing adverse effects and lowering the overall process yield.

**Table 1:** Pretreatment conditions and composition for each lignocellulosic biomass sample

Biomass No	Pressure (bar)	Temperature (°C)	Reaction time (min)
*ACΟ/H*_*2*_*O*			
1^a^	16	160	60
2^a^	16	160	120
3^b^	8	160	120
4^a^	16	175	30
5^a^	16	175	60
6^a^	16	175	120
7^b^	8	175	120
8	16	175	120
*EtOH/H*_*2*_*O*			
9^a^	16	160	60
10^a^	16	160	120
11^b^	8	160	120
12^a^	16	175	60
13^a^	16	175	120
14	8	175	120
*THF/H*_*2*_*O*			
15^a^	16	160	60
16^a^	16	160	120
17	8	160	120
18^a^	16	175	60
19^a^	16	175	120
20	8	175	120

The solvent ratio to water in each pretreatment experiment was 1:1

^a^Kalogiannis et al. [19]

^b^Karnaouri et al. [20]

**Table 2:** Hydrolysis yields and glucose isomerization to fructose for different pretreated biomass samples

Biomass No	Glucose (g/L)		Cellulose to glucose conversion (%)		Glucose to fructose conversion (%)		g fructose/g cellulose		g fructose/100 g pretreated biomass	
*ACΟ/H*_*2*_*O*										
1	59.91	(0.49)	77.98	(0.64)	78.80	(0.14)	0.61	(0.10)	38.00	(1.66)
2	87.78	(2.22)	92.79	(2.34)	79.87	(0.13)	0.74	(0.10)	56.79	(1.34)
3	68.17	(2.15)	82.71	(2.61)	76.34	(0.16)	0.63	(0.16	42.16	(2.36)
4	80.16	(0.22)	81.44	(0.22)	77.23	(0.15)	0.63	(0.13)	50.15	(1.60)
5	71.97	(0.99)	69.99	(0.97)	75.00	(0.17)	0.51	(0.13)	42.38	(1.54)
6	71.58	(0.22)	67.34	(0.21)	73.68	(0.18)	0.50	(0.12)	42.72	(1.42)
7	78.06	(0.99)	76.83	(0.98)	78.58	(0.14)	0.60	(0.10)	49.68	(1.23)
8	42.78	(0.37)	56.25	(0.48)	68.12	(0.21)	0.38	(0.12)	23.61	(1.88)
*EtOH/H*_*2*_*O*										
	33.73	(0.00)	50.39	(0.00)	71.53	(0.00)	0.36	(0.00)	19.55	(0.00)
9	55.38	(0.25)	79.06	(0.35)	74.38	(0.17)	0.59	(0.14)	33.37	(2.45)
10	80.26	(1.18)	89.11	(1.31)	76.30	(0.16)	0.68	(0.13)	49.61	(1.78)
11	48.37	(1.63)	59.02	(1.32)	72.37	(0.68)	0.43	(0.01)	28.36	(1.84)
12	84.59	(1.06)	88.88	(1.11)	75.94	(0.17)	0.64	(0.16)	49.36	(2.03)
13	82.23	(2.65)	80.67	(2.15)	73.13	(0.44)	0.59	(0.02)	48.70	(2.41)
14	86.08	(0.56)	92.21	(0.45)	79.05	(0.09)	0.73	(0.00)	55.15	(0.55)
*THF/H*_*2*_*O*										
15	79.51	(0.12)	93.36	(0.14)	80.11	(0.13)	0.75	(0.13)	51.60	(1.83)
16	78.70	(1.81)	80.57	(1.85)	78.19	(0.14)	0.63	(0.10)	49.85	(1.27)
17	57.60	(0.32)	71.49	(0.40)	74.14	(0.17)	0.53	(0.12)	34.59	(1.84)
18	83.27	(2.58)	79.10	(2.45)	79.12	(0.14)	0.63	(0.09)	53.37	(1.02)
19	78.98	(0.84)	75.09	(0.68)	70.80	(0.23)	0.53	(0.01)	45.28	(0.74)
20	79.47	(1.54)	84.31	(1.24)	78.97	(0.26)	0.67	(0.01)	50.81	(1.51)
Untreated	12.86	(0.19)	25.97	(0.15)	70.89	(0.31)	0.18	(0.00)	7.38	(0.35)
Avicel®	76.05	(0.26)	64.51	(0.21)	80.40	(0.11)	0.50	(0.08)	48.21	(0.81)

Numbers in parenthesis represent the standard error values

All experiments were run in duplicates and numbers represent the mean values

**Fig. 2:**
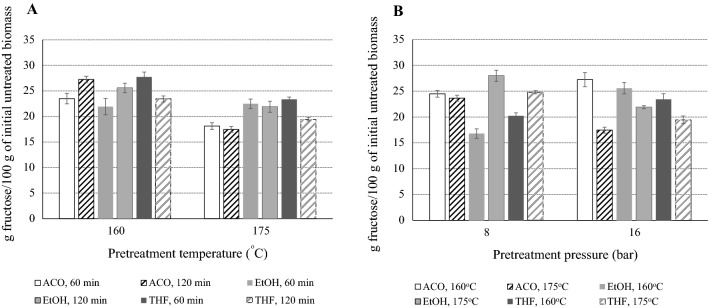
Effect of solvent, temperature and time when pretreatment is operated at O_2_ pressure of 16 bar (**A**) and effect of pressure and temperature when pretreatment is operated for 120 min (**B**) on the overall fructose yield

Correlating the effect of organic solvent in concert with the pretreatment temperature and the residence time with the saccharification yields of the cellulose-rich pulps, fractionation with ACO and THF favored a higher glucose release at 160 °C, compared to 175 °C. A shorter ACO pretreatment (30 min) was more efficient at higher temperature (175 °C), reaching 80.16 g/L glucose (81.44% conversion), while 120 min were required at 160 °C to reach 87.78 g/L glucose (92.79% conversion), respectively. A similar trend was observed with THF and, to a lesser extent, with EtOH. Regarding the isomerization step, glucose conversion to fructose was approximately 70–80% in all different trials, without showing any particular trend among the pretreated samples, possibly due to the increased cellulose content achieved after OxiOrganosolv fractionation and the absence of inhibitors; the fact that isomerization reaction conditions were previously optimized should be also taken into consideration. As shown in Fig. 2, when considering the overall process of pretreatment, hydrolysis and isomerization, it is easy to point out that all solvents perform better when pretreatment occurs at 160 °C compared to 175 °C, while lowering the pressure from 16 to 8 bar has varying effects depending also on the pretreatment temperature. The highest fructose yield obtained with all three solvents is approximately 27 g fructose/100 g of initial biomass and it is achieved when pretreatment occurs at 120 min, 160 °C and 16 bar (ACO), 120 min, 175 °C and 8 bar (EtOH) and 60 min, 160 °C and 16 bar (THF). Considering that direct saccharification and isomerization of untreated biomass results in 7.38 g fructose/100 g of untreated biomass, it is highlighted that OxiOrganosolv pretreatment promotes a fourfold increase of the overall fructose yield.

While there are numerous reports studying the conversion of glucose and/or fructose to HMF presented eloquently in many review papers [22–24], there are fewer reports describing thoroughly the individual process steps of producing HMF from lignocellulosic biomass [25–27], especially by coupling enzymatic and chemo-catalysis, as is the goal of the current work. An early study examining HMF production via fructose reported 90% glucose conversion to fructose in optimal borate loading and a following 63.3% HMF yield during the dehydration step [11]. However, these results report the isomerization of pure glucose and not a hydrolysate obtained after the enzymatic saccharification of a real lignocellulosic substrate. Another work reports similar isomerization experiments on pure sugars to transfer these conditions to real dry matter (DMR) hydrolysates [18]. The isomerization experiments took place in 30 g/L pure glucose, 90 g/L pure glucose and 90 g/L glucose in DMR hydrolysate. The respective fructose yields were 83%, 77% and approximately 75% with the gradual decrease attributed to either the increase of the substrate or the fact that the hydrolysate contains various other substances that could potentially affect the isomerization reaction. In the present work, actual lignocellulosic biomass was used as feedstock. It was pretreated via the OxiOrganosolv process and the pre-treated lignin-free biomass was subjected to enzymatic saccharification followed by enzymatic isomerization of the produced glucose, thus achieving up to 80.11% conversion to fructose, without any other intermediate treatment, purification or separation of the produced liquid streams. This is probably the first work that examines a number of differently pretreated biomasses with regard to their potential to produce fructose, in fact optimally transforming more than half of the pretreated biomass in fructose (56.79 g fructose/100 g pretreated biomass; Table 2).

### Scale up reaction and dehydration to furans

**Fig. 3:**
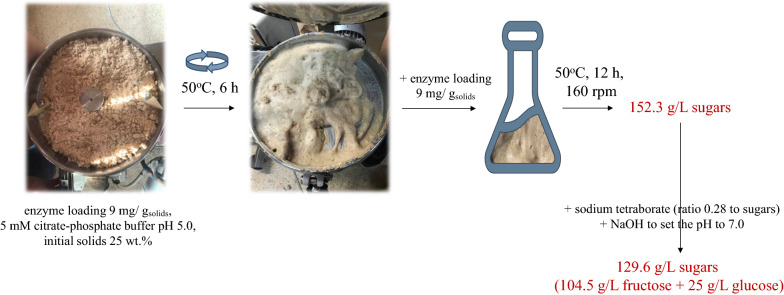
Overall scheme of scale-up reaction towards the production of a fructose-rich syrup

To obtain a concentrated fructose syrup that could serve as a starting material for the production of furans, a scale-up reaction was designed (Fig. 3). The employment of a high-gravity process, namely, with a solids loading higher than 20 wt. % is a prerequisite to obtain highly concentrated sugar syrups. However, it often suffers from poor mass transfer conditions; the adverse effects can be alleviated through a partial enzymatic hydrolysis step, which is referred to as liquefaction [28]. Liquefaction was performed in a two-step process, starting with incubation in a free-fall mixer and subsequent stage in an Erlenmeyer flask, which was proved out to be an efficient strategy to obtain a liquor with 152.3 g/L reducing sugars.

The sugars-rich hydrolysate obtained after isomerization of the scale-up reaction, containing 104.5 g/L fructose and 25 g/L glucose, as well as sodium tetraborate in a ratio to sugars equal to 0.28, was used as a substrate for the production of HMF. Initially, the sugars-rich hydrolysate was diluted with DMSO, a polar aprotic solvent often used in the dehydration reactions for the synthesis of HMF, at a ratio 1 to 4 and was heated at 150 °C for 60 min, according to a previous study by our group [29]. Under the reaction conditions employed, HMF was not obtained as an end-product despite the almost complete conversion of sugars (99.0%) (Additional file 1: Table S2). In fact, the only products identified were organic acids glycolic, acetic and formic, with respective values of yield and selectivity below 4.6%, in all cases (Additional file 1: Table S2). Similar results were obtained in neat H_2_O (i.e. omitting DMSO), as at sugars conversion of 98.9% the products detected were the same organic acids but at respective yield and selectivity below 3.2% (Additional file 1: Table S2). The only difference in this case, was that some traces of HMF were also detected, but with negligible yield (0.5%) and selectivity (0.5%). Thus, it was concluded that certain components of the sugars-rich liquid fraction, originating from pre-treated biomass hydrolysis and glucose isomerization processes, act as catalysts causing conversion of sugars all the way to humins.

Since the conversion of sugars is promoted by acidic ions, it was assumed that either the buffer citrate–phosphate from the hydrolysis step or the sodium tetraborate from the isomerization step might be the culprits causing the undesired conversion of the sugars to humins. Therefore, three ‘model’ hydrolysates were prepared containing (1) glucose (0.5 wt.%), fructose (2.1 wt. %) and buffer citrate–phosphate, (2) glucose (0.5 wt.%), fructose (2.1 wt. %) and sodium tetraborate, (3) glucose (0.5 wt.%), fructose (2.1 wt. %), buffer citrate–phosphate and sodium tetraborate, simulating the composition of the actual sugars-rich hydrolysate diluted with H_2_O at 1 to 4 ratio. In each solution, the pH was adjusted to 7 with NaOH, simulating the actual process and afterwards it was subjected to heating at 150 °C for 60 min. According to the experimental results (Table 3), sodium tetraborate alone proved highly active, as the conversion of sugars reached 99.6%, while HMF yield (0.1%) and selectivity (0.1%) were negligible. On the other hand, citrate–phosphate buffer alone demonstrated mild catalytic activity, with sugars conversion and HMF selectivity reaching low values of 23.1 and 30.2%, respectively. Finally, in the presence of both citrate–phosphate and sodium tetraborate, despite sugars conversion reaching 95.4%, the yield of HMF was barely 0.1%. Based on the results obtained from these ‘model’ hydrolysates, it was concluded that the presence of sodium tetraborate in the sugars-rich hydrolysate, and more specifically of boron species (tetraborate anion, boric acid) generated by its interaction with water, boosts the glucose conversion reactions towards the formation of by-products (humins), as also indicated by the dark brown color of the solution after the reaction (Additional file 1: Figure S2). According to previous studies [30–32], high content of boron species can lead to the formation of a complex with the sugar bound to two borate molecules. These complexes of fructose are likely to be more stable than the mono-borate complexes of fructose, thus blocking the subsequent elimination of water in the first step of the conversion to HMF (proposed reaction mechanism by Ståhlberg et al. [30]) and simultaneously favoring fructose polymerization reactions [33, 34]. The above hypothesis is also reinforced by the negligible production of formic (yield 2.3%) and levulinic (yield 1.5%), valuable organic acids derived from HMF (Table 3).

**Table 3:** Effect of citrate–phosphate buffer and sodium tetraborate addition on model compound sugars (glucose 0.5 wt. % and fructose 2.1 wt. %) conversion to HMF (Reaction conditions: 150 °C, 60 min)

Solvent	Additive	Sugars conversion (%)^#^	HMF Selectivity (%)^#^	Yield (%)^#^			
				HMF	Glycolic acid	Formic acid	Levulinic acid
dH_2_O	Borax*, NaOH	99.6	0.1	0.1	0.9	2.2	1.5
Buffer citrate–phosphate (5 mM)	NaOH	23.1	30.2	7.0	0.2	0.2	0.1
Buffer citrate- phosphate (5 mM)	Borax*, NaOH	95.4	0.1	0.1	1.0	2.3	1.5

All experiments were run in duplicates and numbers represent the mean values

^*^sodium tetraborate decahydrate

^**#**^Standard error deviation was ≤ 2.5% in all measurements

Considering the necessity of both the presence of sodium tetraborate and citrate–phosphate buffer for the efficient enzymatic hydrolysis and isomerization of glucose to fructose, efforts were directed towards identifying the proper acid for decomposition of sodium tetraborate to minimize its effect on the production of HMF. Using this approach, Huang et al. [11] reported 63% yield and 71.8% selectivity of HMF, with 88% conversion of sugars, performing the reaction in the presence of HCl, in water/MIBK diphasic system. In addition, using the same inorganic acid (HCl), but different biphasic system (dioxane:H_2_O equal to 2:1), Wang et al. [18] reported even higher HMF yield (75%) and selectivity (88%), while the corresponding values using aqueous solution of H_2_SO_4_ (0.1 M) were 39% and 57%, based on the study of Rivas et al. [35]. Under strong acidity provided by HCl or H_2_SO_4_, sodium tetraborate is decomposed to boric acid and the corresponding sodium salt. Boric acid catalyses the formation of HMF; however, it also enables polymerization side reactions, as mentioned by the Istasse et al. [31], especially in the presence of salts as demonstrated by Hansen et al. [36]. Apart from the boron species, another crucial factor for the selective synthesis of HMF is pH. It was demonstrated [37] that in acidic pH below 2.0, the formation of HMF is favored, contrary to higher pH values, where sugars decomposition products, such as organic acids and humins, are enhanced. Based on these and our results, as presented in Table 3, the coupling of enzymatic and chemocatalytic processes requires the addition of an acid to convert the borate ions to boric ions and simultaneously act as a catalyst for the dehydration reaction. Thus, the effect of two strong inorganic (HCl and H_3_PO_4_) and two milder organic acids (formic and maleic) was examined, as presented in Table 4. Furthermore, the effect of a solid acid catalyst, namely, H-mordenite, was examined without prior acidification of the solution.

**Table 4:** Catalyst evaluation for HMF synthesis from sugars (glucose 0.5 wt.% and fructose 2.1 wt. %), in the liquid product after isomerization (Reaction conditions: 150 °C, 60 min)

Catalyst (final concentration wt. %)^#^	Sugars conversion (%)^#^	HMF yield (%)^#^	HMF selectivity (%)^#^
HCl (0.7%)	76.1	36.3	47.7
H_3_PO_4_ (3.0%)	83.1	43.7	52.6
Formic acid (3.5%)	79.9	44.6	55.8
Maleic acid (3.0%)	75.4	41.8	55.5
H-mordenite (2.6%)	97.6	1.4	1.4

All experiments were run in duplicates and numbers represent the mean values

^**#**^Standard error deviation was ≤ 2.5% in all measurements

**Fig. 4:**
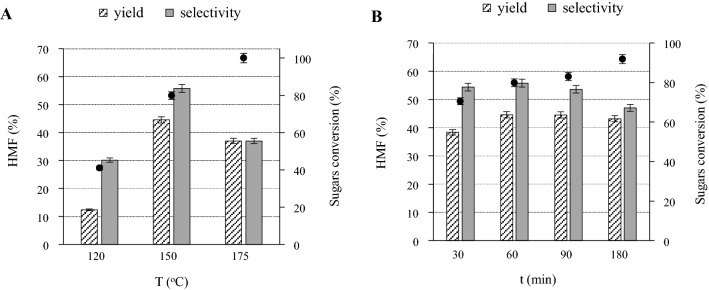
Effect of reaction (**A**) temperature (t: 60 min) and (**B**) time (Τ: 150 °C) on HMF synthesis from hexoses (glucose 0.5 wt. % and fructose 2.1% wt. %) with formic acid catalyst, in the liquid product after isomerization (formic acid 3.5 wt. %). (Primary *y* axis (bars): % HMF yield and selectivity, Secondary *y* axis (bullets): sugars conversion)

Thus, sugars-rich hydrolysate was acidified by addition of concentrated HCl, H_3_PO_4_, formic or maleic acid until pH 1–2, to ensure the dissociation of the borate species followed by dilution with H_2_O at a ratio 1 to 4. Afterwards, the acidified and diluted hydrolysate was treated at 150 °C for 60 min. According to the experimental results (Table 4), both the inorganic and the organic acids exhibited similar performance, thus catalyzing fructose conversion to HMF within a range of 75.4–83.1%, reaching yield between 36.3–44.6% and a selectivity between 47.7–55.8%. Compared to the results in the absence of any catalyst, these results validate the need for inactivating sodium tetraborate to boost the synthesis of HMF and suppress the subsequent decomposition reactions. Other products detected were organic acids formic and levulinic (Additional file 1: Table S3), but with respective yields below 3.8 and 2.6% in all cases, except the one, where formic acid was used as catalyst. In that case, the concentration of the produced formic acid could not be calculated, while the yield and selectivity of levulinic acid reached the highest values of 10.0 and 12.6%, respectively. In fact, among the homogeneous catalysts tested, formic acid presented the best results with HMF yield and selectivity reaching values of 44.6 and 55.8%, respectively, while sugars conversion was 79.9%. Furthermore, to evaluate the possibility of applying heterogeneous catalysis, H-mordenite was added to the sugars-rich hydrolysate, followed by treatment at 150 °C for 60 min. However, it did not have a discernible effect on HMF synthesis probably due to the presence of borate ions, as explained above. Since formic acid offered the highest HMF yield and selectivity, it was studied further regarding the effect of the reaction conditions on the synthesis of HMF from the sugars-rich hydrolysate. Thus, sugars-rich hydrolysate acidified by formic acid, was treated at three levels of temperature ranging from 120 to 175 °C and four different levels of reaction time ranging from 30 to 180 min. The results (Fig. 4) indicated that sugars (i.e. both glucose and fructose) conversion increased with temperature and time, while HMF selectivity and yield presented a maximum at 150 °C for 60 min (55.8 and 44.6%, respectively). At temperatures higher than 150 °C and longer reaction times, HMF selectivity and yield decrease, likely due to the formation of unidentified products (humins), as indicated form the dark brown color of the reaction solution. Consequently, the optimum reaction conditions for HMF selectivity and yield, are 150 °C for 60 min of reaction time. Overall, HMF can be produced from the sugars-rich hydrolysate at 45% yield using formic acid for alleviating the detrimental effect of borate species on the dehydration of sugars. The proposed process starting from actual lignocellulosic biomass results in the synthesis of HMF at satisfactory yields, while it is performed in aqueous solutions, without intermediate separation steps and omitting the use of extreme reaction conditions and inorganic acids, thus complying with the principles of green chemistry and being cost-effective.

## Conclusions

Lignocellulose has always been in the spotlight as a candidate for the production of 2G biofuels. However, over the last few years there have been attempts to transform lignocellulose into other high added value products, among others being furans and biobased monomers. Towards this concept, this work presents a combination of processes to effectively transform lignocellulosic biomass to HMF, including pretreatment, enzymatic saccharification and isomerization as well as chemical dehydration of fructose to HMF. Ultimately, the fact that more than half of lignocellulose was converted to fructose and the subsequent high yield and selectivity of HMF, support the feasibility of the entire process. Future work includes the design and production of suitable enzymes that can effectively catalyze the oxidation of HMF. This way, a potential integrated process starting from lignocellulose and leading to biobased monomers can be feasible.

## Materials and methods

### Organosolv pretreatment of beechwood biomass

The lignocellulosic material that was used as a feedstock in this study represents beechwood biomass (Lignocel® HBS 150–500, JRS GmbH and Co KG, Germany). The samples were subjected to mild oxidative organosolv pretreatment (OxiOrganosolv) according to Kalogiannis et al. [19]. Different organic solvents were used, including acetone (ACO), ethanol (EtOH) and tetrahydrofuran (THF), in aqueous solutions of 50% (water to organic solvent ratio 1:1), in two different residence times (60, 120 min). Pretreatment process was initiated after pressurizing 100% O_2_ in the vessel by heating up to the pretreatment temperature (160, 175 °C). At the end of the process, the slurry was vacuum filtered; the solid part was received, washed with organic solvent and distilled water until pH of 5.0 and it was air-dried. The composition of the untreated biomass was 23.6 wt. % lignin, 40.1 wt. % cellulose and 19.1 wt. % hemicellulose [19]. Compositional analysis of pretreated biomass was identified according to NREL protocols [38].

### Hydrolysis of pretreated solid pulps and glucose isomerization to fructose

To evaluate the effect of different pretreatment conditions on enzymatic depolymerization and production of glucose, hydrolysis experiments were conducted in 250 mL Erlenmeyer flasks, upon addition of Cellic® CTec2 (Novozymes A/S, Denmark), at an enzyme loading of 15 mg/g_solids_. Filter paper activity and protein content of Cellic® CTec2 were found to be 168 FPU/mL (FPU: filter paper units) and 137 mg/mL, respectively, using the methods described by Ghose [39] and Bradford [40]. The initial solids content was 10 wt. % and the reactions occurred for 72 h, at 100 mM citrate–phosphate buffer pH 5.0, under agitation (160 rpm). Total reducing sugars after the hydrolysis experiments were determined according to the dinitro-3,5-salicylic acid (DNS) method [41], while glucose was measured using the commercial enzyme preparation of glucose oxidase/peroxidase assay (GOD/PAP, Biosis, Greece).

For the isomerization step, the immobilized glucose isomerase Sweetzyme® was used (Novozymes A/S, Denmark). The difference in the pH optimal values of the hydrolytic enzymes and the glucose isomerase, prevented the one-pot reaction approach for the production of fructose from the cellulose-rich solid fraction after pretreatment. Hence, the isomerization step took place after pH of the hydrolysate was adjusted to 7.0 upon addition of NaOH 1 M. Sodium tetraborate decahydrate (Borax decahydrate) was added to shift the equilibrium in favor of fructose formation. Preliminary experiments to determine the optimum concentration of sodium tetraborate that leads to increased isomerization rates was conducted with pure glucose at an initial substrate concentration of 60 g/L, at 50 °C, in a citrate–phosphate buffer pH 5.0 adjusted to 7.0 with NaOH 1 M. The glucose isomerase loading was 5% w/v. Sodium tetraborate to glucose molar ratio tested was within the range of 0.05–0.5. After identifying the optimal conditions for the reaction, a similar procedure was followed for the isomerization of the biomass-derived hydrolysates, by adding the appropriate amount of sodium tetraborate. It is noted that the volume of NaOH added to each hydrolysate was too small to cause a significant reduction of the glucose concentration at this step. Since the only product of the glucose isomerization is fructose, the respective fructose yields were calculated by the reduction of the glucose concentration at the end of the reaction, after filtration of the samples.

### Scale-up hydrolysis/isomerization reaction for the production of high-fructose syrup

Scale-up reaction hydrolysis (800 mL) of the biomass pretreated with EtOH, at 175 °C for 60 min took place in two steps. The first occurred in a custom-made free fall mixer [42], where the initial solids loading was 25% wt., with an enzyme loading of 9 mg/g_solids_, in 5 mM citrate–phosphate buffer pH 5.0, at 50 °C, for 6 h. The second step involved the addition of extra enzyme until a final loading of 18 mg/g_solids_ and the hydrolysis took place in an Erlenmeyer flask in a volume of 800 mL, under agitation 160 rpm at 50 °C for 12 h. At the end of hydrolysis, the slurry was vacuum filtrated and the sugar-rich hydrolysate was subjected to isomerization with Sweetzyme®, as described above, by adding the appropriate amount of sodium tetraborate and changing the pH value to 7.0 with NaOH. The obtained liquor was used as a substrate for the catalytic production of furans, as described below.

### Dehydration reaction of monomeric sugars towards their conversion to furans

The sugar-rich liquor resulting from the enzymatic hydrolysis and isomerization of biomass (glucose 25 g/L and fructose 104.5 g/L) was converted to furans over homogenous and/or heterogeneous catalysts. Thus, HCl 37 wt. %, H_3_PO_4_ 85 wt.%, formic acid 99 wt.% and maleic acid (solid form) used as homogenous catalysts, were purchased from Sigma Aldrich and used as received. Zeolite H-mordenite, employed as a heterogenous catalyst, was prepared via calcination of the NH_4_-form zeolite CBV 21A (Si/Al = 10) (Zeolyst International) at 500 °C for 3 h in air. The experiments were carried out in a batch, stirred, autoclave reactor (C-276 Parr Inst., USA), under N_2_ gas. The sugar-rich liquor was diluted with water, to suppress side reactions and increased formation of by-products (dilution factor 1/4) and the corresponding catalyst was added. The mixture was charged into the reactor and heated to the desired temperature. The reaction was allowed to proceed for a given time under continuous stirring. After completion, the reactor was cooled swiftly; the solution was filtered to remove solids (i.e. catalyst, by-products etc.) and analyzed by Ion Chromatography (ICS-5000, Dionex, USA). The quantification was based on external calibration, using standard solutions of sugars (glucose, mannose, xylose, fructose, galactose, arabinose and rhamnose), sugar alcohols (sorbitol and mannitol), hydroxymethylfurfural (HMF) and organic acids (formic, acetic, glycolic, lactic, levulinic, propionic and butyric acid). The analysis of sugars was performed using a CarboPac PA1 (10 μm, 4 × 250 mm) column and guard column (10 μm, 4 × 30 mm) connected to a pulsed amperometric detector (PAD). The eluent was 20 mM NaOH at a 0.6 ml/min flow rate and the total analysis time was 75 min. The analysis of the organic acids was performed on an AS-15 (9 μm, 4 × 250 mm) column and pre-column (9 μm, 4 × 30 mm) connected to a conductivity detector (CD). The eluent was 8 mM NaOH at a 1 ml min^−1^ flow rate and the total analysis time was 75 min.

The conversion of sugars (glucose and fructose), the yields and the selectivity of the products (weight based) were calculated according to the following Eqs. (1), (2) and (3):

1 $$\text{Conversion}_{\text{sugars}} \left( \% \right) = 100 \times \frac{\text{sugars}\; \text{reacted}\;g}{{\text{sugars}\; \text{initial} \;g}}$$

2 $$\text{Yield}_{\text{product}} \left( \% \right) = 100 \times \frac{\text{product}\; \text{produced} \;g}{{\text{sugars}\; \text{initial}\; g}}$$

3 $$\text{Selectivity}_{\text{product}} \left( \% \right) = 100 \times \frac{{\text{Yield}_{\text{product}} \left( \% \right)}}{{\text{Conversion}_{\text{sugars}} \left( \% \right) }}$$

## Supplementary Information

**Additional file 1:Figure S1.** Study for the optimum solids and enzyme loading conditions. **Figure S2.** The color gained by the solutions after: **A** blank experiments in **a** H2O and **b** DMSO:H2O (4:1) mixture and **B** in pure aqueous solution of sugars in the presence of (a) sodium tetraborate decahydrate and NaOH, (b) buffer citrate phosphate (5 mM) and NaOH and (c) sodium tetraborate decahydrate, buffer citrate-phosphate (5 mM) and NaOH. **Table S1.** Composition for each lignocellulosic biomass sample. **Table S2.** Sugars (glucose and fructose, 2.5 wt. %) conversion to HMF in the absence of any additional catalyst (150 °C, 60 min). **Table S3.** Effect of homogeneous and heterogeneous catalysts on the decomposition reactions of sugars (glucose and fructose 2.5 wt. %) to organic acids (150 °C, 60 min).

## Data Availability

Organosolv pretreated beechwood biomass, as well as high-fructose syrup from scale-up reaction are available upon reasonable request.
