# Supplementary material for: Conversion of organosolv pretreated hardwood biomass into 5-hydroxymethylfurfural (HMF) by combining enzymatic hydrolysis and isomerization with homogeneous catalysis
Source: Biotechnol Biofuels. 2021 Aug 28;14:172. doi: 10.1186/s13068-021-02022-9 (PMC8403452; doi:10.1186/s13068-021-02022-9)
Supplement: Supplementary file 1 — Additional file 1:Figure S1. Study for the optimum solids and enzyme loading conditions. Figure S2. The color gained by the solutions after: A blank experiments in a H2O and b DMSO:H2O (4:1) mixture and B in pure aqueous solution of sugars in the presence of (a) sodium tetraborate decahydrate and NaOH, (b) buffer citrate phosphate (5 mM) and NaOH and (c) sodium tetraborate decahydrate, buffer citrate-phosphate (5 mM) and NaOH. Table S1. Composition for each lignocellulosic biomass sample. Table S2. Sugars (glucose and fructose, 2.5 wt. %) conversion to HMF in the absence of any additional catalyst (150 °C, 60 min). Table S3. Effect of homogeneous and heterogeneous catalysts on the decomposition reactions of sugars (glucose and fructose 2.5 wt. %) to organic acids (150 °C, 60 min). [file 13068_2021_2022_MOESM1_ESM.docx]

**Conversion of organosolv pretreated hardwood biomass into 5-hydroxymethylfurfural (HMF) by combining enzymatic hydrolysis and isomerization with homogeneous catalysis**

**Grigorios Dedes, Anthi Karnaouri, Asimina A. Marianou, Konstantinos G. Kalogiannis, Chrysoula M Michailof, Angelos A. Lappas, Evangelos Topakas**

**Supplementary Material**


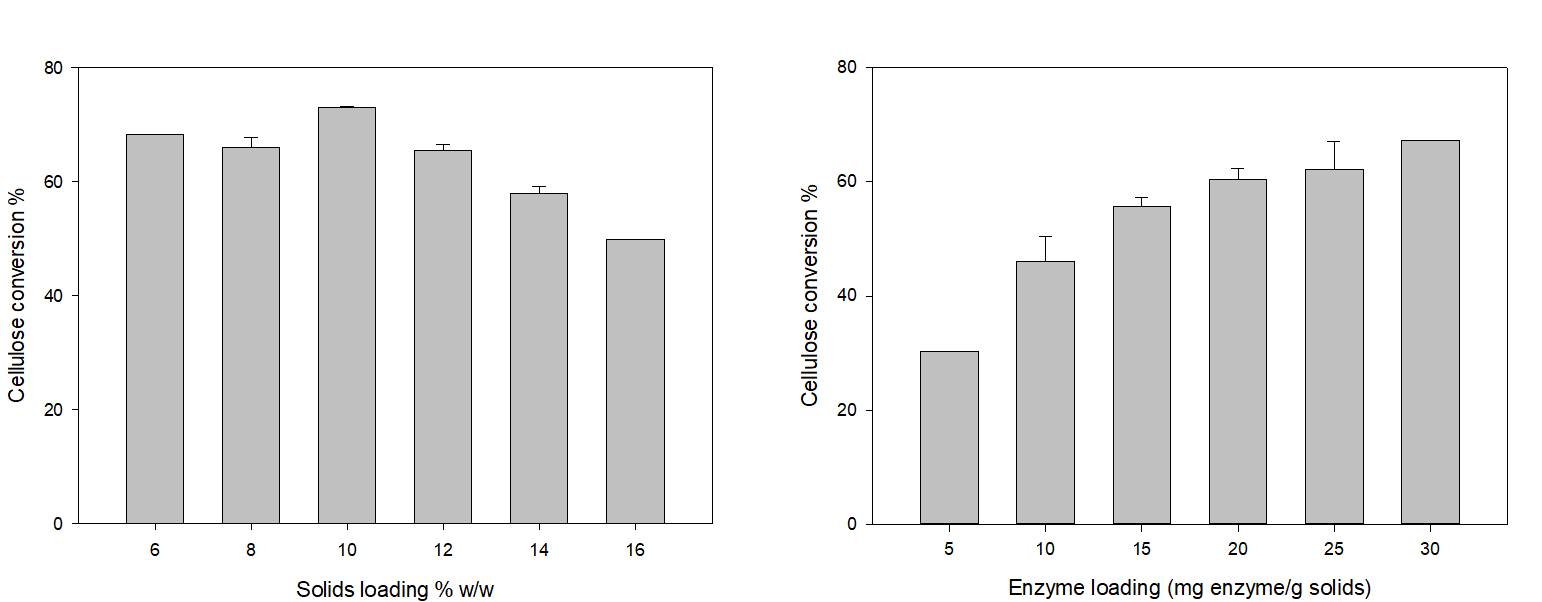


**Figure S1.** Study for the optimum solids and enzyme loading conditions

**
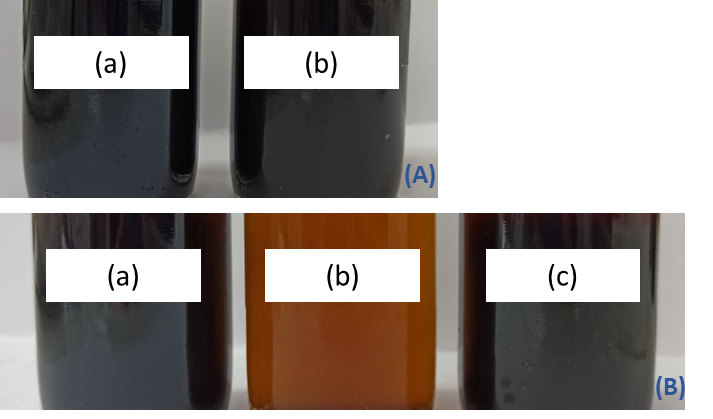
**

**Figure S2.** The color gained by the solutions after: **(A)** blank experiments in **(a)** H_2_O and **(b)** DMSO:H_2_O (4:1) mixture and **(B)** in pure aqueous solution of sugars in the presence of **(a)** sodium tetraborate decahydrate and NaOH, **(b)** buffer citrate phosphate (5mM) and NaOH and **(c)** sodium tetraborate decahydrate, buffer citrate-phosphate (5mM) and NaOH.

**Table S1**. Composition for each lignocellulosic biomass sample.

| **Biomass No.** | **Lignin (%)** | **Cellulose (%)** | **Hemicellulose (%)** |
| --- | --- | --- | --- |
| *ACΟ/H_2_O* |  |  |  |
| 1^a^ | 13.7 | 62.2 | 20.2 |
| 2^a^ | 3.2 | 76.6 | 13.3 |
| 3^b^ | 10.5 | 66.8 | 18.4 |
| 4^a^ | 3.6 | 79.7 | 15.7 |
| 5^a^ | 1.2 | 83.3 | 15.3 |
| 6^a^ | 1.6 | 86.1 | 8.7 |
| 7^b^ | 4.6 | 82.3 | 13.9 |
| 8 | 12.3 | 61.6 | 19.2 |
|  |  |  |  |
| *EtOH/H_2_O* |  |  |  |
| 9^a^ | 16.2 | 56.8 | 20.0 |
| 10^a^ | 6.4 | 73.0 | 16.0 |
| 11^b^ | 10.5 | 66.4 | 21.0 |
| 12^a^ | 2.7 | 77.1 | 15.7 |
| 13^a^ | 2.1 | 82.6 | 14.5 |
| 14 | 3.9 | 75.6 | 16.4 |
|  |  |  |  |
| *THF/H_2_O* |  |  |  |
| 15^a^ | 11.4 | 69.0 | 15.5 |
| 16^a^ | 5.5 | 79.1 | 12.1 |
| 17 | 14.9 | 65.3 | 15.0 |
| 18^a^ | 5.1 | 85.3 | 10.8 |
| 19^a^ | 2.6 | 85.2 | 10.6 |
| 20 | 6.5 | 76.4 | 13.6 |

^a^Results adapted from previous work (Kalogiannis et al., 2020)

^b^Results adapted from previous work (Karnaouri et al., 2020)

**Table S2.** Sugars (glucose and fructose, 2.5 wt. %) conversion to HMF in the absence of any additional catalyst (150 ^o^C, 60 min).

| **Solvent** | **Sugars conversion (%)** | **HMF Selectivity (%)** | **Yield (%)** | | | | |
| --- | --- | --- | --- | --- | --- | --- | --- |
|  |  |  | **HMF** | **Glycolic acid** | **Acetic acid** | **Formic acid** | **Levulinic acid** |
| H_2_O | 98.9 | 0.5 | 0.5 | 1.8 | - | 3.1 | 2.1 |
| DMSO/H_2_O (4/1) | 99.0 | - | - | 1.4 | 4.6 | 3.2 | - |

**Table S3**. Effect of homogeneous and heterogeneous catalysts on the decomposition reactions of sugars (glucose and fructose 2.5 wt. %) to organic acids (150 ^o^C, 60 min).

| **Catalyst (final concentration wt.%)** | **Yield (%)** | | **Selectivity (%)** | |
| --- | --- | --- | --- | --- |
|  | **Formic acid** | **Levulinic acid** | **Formic acid** | **Levulinic acid** |
| HCl (1.5%) | 3.8 | 2.6 | 5.0 | 3.4 |
| H_3_PO_4_ (3.1%) | 2.3 | 1.6 | 2.8 | 1.9 |
| formic acid (3.5%) | - | 10.0 | - | 12.6 |
| maleic acid (3%) | 3.0 | 2.0 | 4.0 | 2.7 |
| H-Mordenite (2.6%) | 3.3 | 2.2 | 3.4 | 2.3 |
